# Spatially Filtered Multilevel Analysis on Spatial Determinants for Malaria Occurrence in Korea

**DOI:** 10.3390/ijerph16071250

**Published:** 2019-04-08

**Authors:** Sehyeong Kim, Youngho Kim

**Affiliations:** 1 Department of Geography, Korea University, 145 Anam-ro, Seoul 02841, Korea; kseh92@gmail.com; 2 Department of Geography Education, Korea University, 145 Anam-ro, Seoul 02841, Korea

**Keywords:** malaria, multilevel model, eigenvector spatial filtering

## Abstract

Since its re-emergence in 1993, the spatial patterns of malaria outbreaks in South Korea have drastically changed. It is well known that complicated interactions between humans, nature, and socio-economic factors lead to a spatial dependency of vivax malaria occurrences. This study investigates the spatial factors determining malaria occurrences in order to understand and control malaria risks in Korea. A multilevel model is applied to simultaneously analyze the variables in different spatial scales, and eigenvector spatial filtering is used to explain the spatial autocorrelation in the malaria occurrence data. The results show that housing costs, average age, rice paddy field ratio, and distance from the demilitarized zone (DMZ) are significant on the level-1 spatial scale; health budget per capita and military base area ratio are significant on the level-2 spatial scale. The results show that the spatially filtered multilevel model provides better analysis results in handling spatial issues.

## 1. Introduction

**Figure 1:**
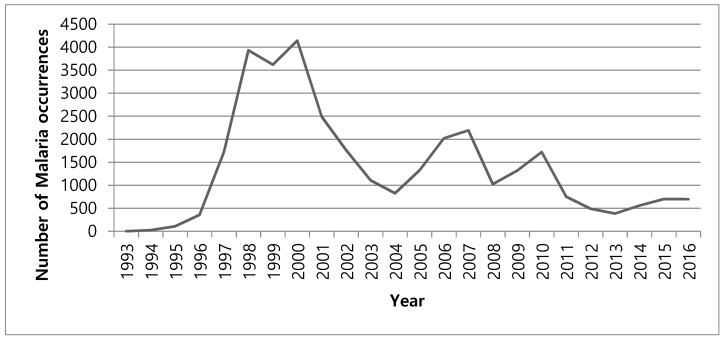
Number of malaria occurrences in Korea since its re-emergence.

*Plasmodium vivax*, an endemic factious disease known as tertian malaria, was prevalent on the Korean peninsula for centuries. It was eradicated in South Korea in 1979 through a national control project and improvements in public sanitation [1]. However, a military serviceman in Paju brought about its re-emergence in 1993. In the following years, the number of malaria occurrences peaked in 2000 with 4000 and declined afterward. Since 2010, the number has fluctuated around 1000 cases every year (Figure 1). The transmission of malaria and its occurrences reflect various social and economic conditions such as human demographic changes, the economic situation, and the level of public sanitation in a country [2,3]. The re-emergence of malaria in South Korea is noteworthy because it is speculated to have originated from adjoining North Korea [4,5]. North Korea experienced severe economic aggravation in the 1990s and had severe malaria outbreaks. As malaria is transmitted by mosquitoes, the spatial patterns of malaria occurrence have similar counts in nearby areas, consequently leading to spatial clustering or spatial dependency [6,7,8]. As previously noted, the initial re-emergence occurred in Paju, which is a remote rural area with little population except for several military bases because of its proximity to the border between North and South Korea. As a result, malaria occurrence in South Korea not only reflects socio-economic environments but also presents political and military tensions between the two Koreas.

This study applied multilevel modeling to use variables of multiple levels from a hierarchical structure in analyzing malaria occurrences, which reflects many complicated natural, socio-economic, and political dimensions. Using the multilevel model helps to supplement the limited availability of health and disease data. Data from multiple levels of the spatial scale extend the analysis range and enable further understanding of malaria occurrences. Many multilevel models have analyzed the determinants of malaria [9,10,11,12,13]. However, study targets and areas were mostly limited to *Plasmodium falciparum* in sub-Saharan Africa, the epidemiological characteristics of which are different from vivax malaria in the temperate climate region. Several multilevel modeling methods have been applied to explain the *vivax* malaria in Asia [14,15]. However, they did not account for spatial autocorrelation in the model. A multilevel study analyzed malaria in South Korea [16]. However, neither spatial autocorrelation nor a political perspective of disease was appropriately considered.

Most multilevel modeling assumes that observations are independent of each other [17]. However, this is not a realistic assumption for a malaria analysis. As malarias are transmitted by mosquitoes, the movement of mosquitoes naturally leads to spatial autocorrelation in malaria occurrences. After malaria has emerged, its transmission through mosquitoes becomes exacerbated with human influence as a host. In the process, an infected neighbor will have a greater chance of infecting people due to the malaria transmission cycle. In addition, environmental factors such as swamps and the rice paddy fields in this study have a huge influence in providing breeding habitat for mosquitoes. These factors all contribute to spatial autocorrelation in malaria occurrences.

These types of spatial dependence issues have been widely introduced in the literature [18]. Thus, the application of ordinary multilevel modeling for the analysis of malaria occurrences has limitations because it typically assumes independent and identical distribution (i.i.d.) of the occurrence data. However, most malaria occurrences are spatially dependent, and a corresponding application of ordinary multilevel modeling is non-spatial and unrealistic, which leads to biased or inefficient results [19,20].

A spatial statistical method that handles spatial autocorrelation in spatial data is necessary for reliable spatial data analysis in multilevel modeling. Griffith suggested spatial filtering methods in the hierarchical generalized linear model in the disease mapping of West Nile Virus spread by mosquitoes [21]. Park and Kim applied a spatially filtered multilevel model in the analysis of neighborhood-level health status in South Korea [22].

This study analyzes malaria occurrences in South Korea in 2014 using the multilevel generalized linear model (MGLM). In the analysis, an eigenvector spatial filtering model is applied and accounts for spatial autocorrelation in the model. The results show that malaria transmission in South Korea is significantly influenced by housing costs, average age, rice paddy field ratio, distance from the demilitarized zone (DMZ), health budget per capita, and military base area ratio throughout multiple levels of analysis. This study reveals the unique socio-political conditions on the Korean peninsula related to malaria occurrences. The distance from the DMZ and military base area ratio variables reflect the influence of military tensions and confrontations between North and South Korea. This study helps to extend understanding of local political effects on epidemiology.

## 2. Study Area, Data, and Methodology

### 2.1. Study Area and Data

**Figure 2:**
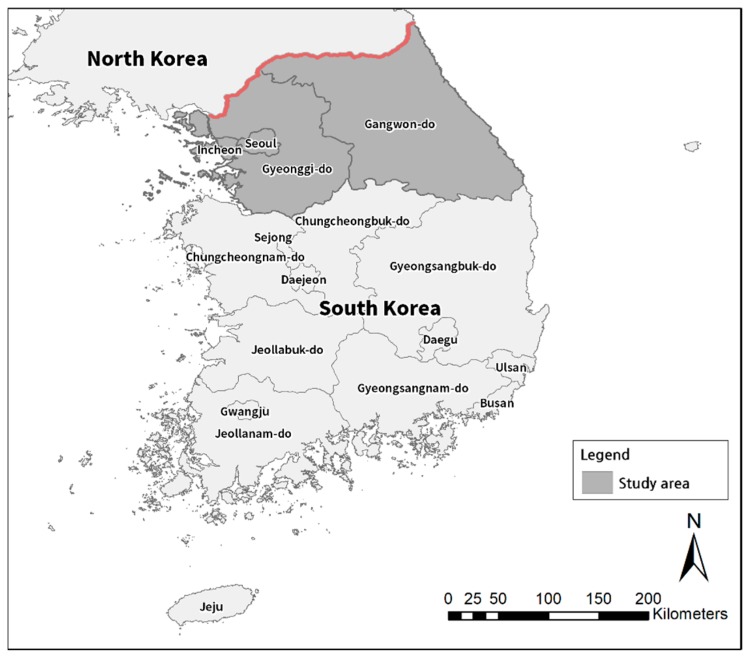
Study area of the research.

Figure 2 shows the study areas composed of Si, Gun, and Gu (city and county) in Seoul, Incheon, Gyeonggi, and Gangwon provinces in South Korea. In total, the data of 1301 districts from 83 cities are used for the modeling. Malaria occurrences in 2014 were obtained from the Korea Centers for Disease Control and Prevention. Descriptive statistics and references for the variables used in the analysis are presented in Table 1.

**Figure 3:**
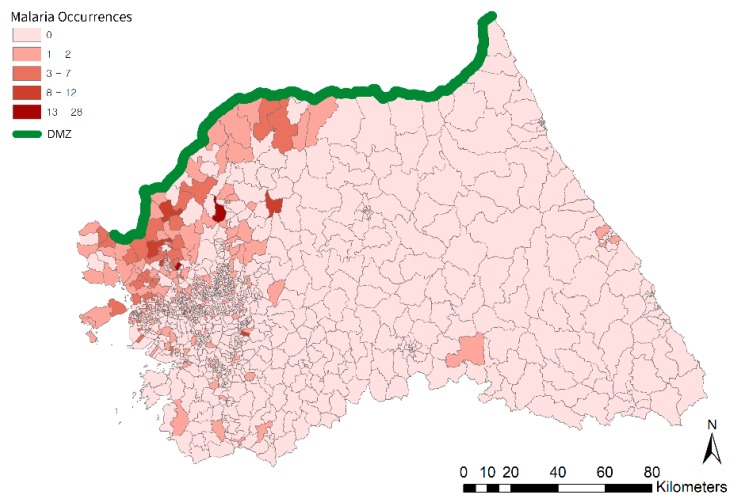
Map of malaria occurrences in South Korea in 2014.

This research uses count data for malaria occurrences as the dependent variable. The point-based case data are aggregated based on administrative areal units such as Eup, Myeon, and Dong (districts, or level-1 scale) as shown in Figure 3. For the analysis at the upper level, the counts at level-1 are aggregated again based on Si, Gun, and Gu (city and county, or level-2). In the literature, disease data are often analyzed by incidence rate standardized by the population, age, or sex. However, this research uses the count data for each areal unit. Due to the epidemiological characteristics in Korea, the use of incident rate per population would be erroneous, leading to a biased analysis result. Military servicemen are not counted in the census population data. Because these servicemen are quite prone and vulnerable to malaria infection, using census data for population would lead to a biased result, especially in this small administrative area. To avoid this bias and distortion in the analysis, malaria occurrence count data are used for the analysis.

Like other infectious diseases, malaria transmission is affected by socio-economic factors [23]. Independent variables in this analysis are composed of socio-economic, demographic, and land use data. The district level (level-1) variables consist of housing costs, sex ratio, average age, rice paddy area ratio, and distance from the DMZ. Obtained from the Ministry of Land, Infrastructure and Transport of Korea, the housing cost is applied as a proxy variable to present the economic status of the area [2]. Sex ratio data were collected from the Population and Housing Census data of Statistics Korea. The sex ratio and age variables present local socio-demographic features of malaria transmissions in South Korea. From its re-emergence until 1997, more than 90% of malaria cases occurred in military soldiers and veterans. From 1997 until today, these soldiers and veterans still account for about half of malaria occurrences in Korea [24,31]. Given that all Korean males must provide military service for two years in their early 20s, sex and age variables are highly relevant in estimating malaria occurrences in Korea. The rice paddy field ratio data are calculated from the land use map provided by the Environmental Geographic Information Service of the Ministry of Environment of Korea in 2013. Anopheline mosquitoes, vectors of malaria, are known to lay their eggs in stagnant water, including rice paddies, ditches, and ponds. As these places are appropriate environments for mosquitoes’ breeding and growing, rice paddy field ratio data are important in estimating malaria occurrences [2]. Distances from the DMZ data are used in this research to evaluate the amount of influence from North Korea and the DMZ region. As Figure 3 shows, given that spatial patterns of malaria occurrences are clustered within the vicinity of the DMZ [7], the re-emergence of malaria in South Korea would have derived from North Korea. The mosquitoes from North Korea with malaria pathogens can pass through the DMZ to South Korea, seeking prey and breeding sites, and such infiltration is assumed to have resulted in the outbreak of malaria occurrences in South Korea [24,26].

The city and county level (level-2) variables are health budget per capita and military base area ratio. Obtained from Statistics Korea, health budget per capita was used to measure the spatial disparities of health care services. The military base area is an important variable in malaria analysis in South Korea because malaria re-emergence was initiated from a military serviceman. In addition, many civilian patients are recently discharged veterans who had been stationed in the nearby DMZ area, causing the spatial diffusion of malaria. Nonetheless, the exact number of military personnel stationed in the study area is not available due to its confidentiality. Instead, the proportion of the military base area is used as a proxy variable for the population of military servicemen. The military base area data were obtained from the National Defense Committee of the National Assembly of Korea in October 2016.

### 2.2. Analysis Methods

#### 2.2.1. Multilevel Model

Multilevel regression method is applied to account for the effects of the level-2 scale on the nested level-1 scale in the data using a hierarchical structure. Spatial data sets in social sciences often have a multilevel structure in that they are collected or measured in multiple spatial scales. The multilevel model assumes that individuals in an upper hierarchy are correlated with each other, and each group is independent of each other. As a result, interaction or contextual effects may exist between different levels. Considering the hierarchical structure of the data, which can potentially result in errors in an ordinary least squares (OLS) model, a multilevel regression is necessary for a better estimation result.

In this research, the malaria occurrence count data are utilized as the dependent variable. Aggregated by the administrative areas, the count data are assumed to follow the Poisson distribution [32,33]. Since the lower bound of the dependent variable is 0, application of the linear regression model has a structural limitation [34]. MGLMs are applied to analyze the non-normal data considering the random effects [35]. MGLMs normalize the dependent variable. The standard link function for the level-1 dependent variable is given by $\log\left(Y_{ij}\right)$, and it is converted back by ${\hat{Y}}_{ij}=\exp(\log({\hat{Y}}_{ij}$)) after the model estimation.

The multilevel modeling begins by analyzing a null model that only contains the hierarchical structure with no independent variables. As level-1 and level-2 independent variables are added to the null model, a multilevel model with all the independent variables from two spatial scales is estimated. The level-2 model integrates the hierarchical variables into one model. The overall model is expressed as follows:

(1) $$\begin{array}{cc} \mathrm{Level}\;1: & \log\left(Y_{ij}\right)=\beta_{0j}+\beta_{10}X_{ij}+e_{ij}\\ \mathrm{Level}\;2: & \beta_{0j}=\gamma_{00}+\gamma_{01}Z_{j}+u_{0j}\\ \mathrm{Combined}: & \log\left(Y_{ij}\right)=\gamma_{00}+\beta_{10}X_{ij}+\gamma_{01}Z_{j}+u_{0j}+e_{ij}, \end{array}$$

where $X_{ij}$ and $Z_{j}$ indicate the independent variables in level-1 and level-2: $X_{ij}$ is the variable in the District level, and $Z_{j}$ is the variable in the County level; $e_{ij}$ is the error term at level-1; $\gamma_{00}$ is a mean of dependent variable $\log\left(Y_{ij}\right)$ after accounting for County-level variables; and $u_{0j}$ is the error term in the County level. In the combined equation, $\gamma_{00}+\beta_{10}X_{ij}+\gamma_{01}Z_{j}$ are fixed effects. $u_{0j}+e_{ij}$ are random effects. In the modeling framework, the intercept is assumed to be inconsistent when there is a large between-group variance.

#### 2.2.2. Spatially Filtered Multilevel Model

Extending from Equation (1), the multilevel model combined with eigenvector spatial filtering is conceptualized as follows:

(2) $$\begin{array}{ll} \log\left(Y_{ij}\right) & =\underset{fixed\;effect}{\underset{︸}{\gamma_{00}+\beta_{10}X_{ij}+\gamma_{01}Z_{j}}}+\underset{random\;effect}{\underset{︸}{u_{0j}+e_{ij}}}\\ & =\underset{fixed\;effect}{\underset{︸}{X\beta \left(=\gamma_{00}+\beta_{10}X_{ij}+\gamma_{01}Z_{j}\right)}}+\underset{random\;effect}{\underset{︸}{\underset{spatial\;filtering\;component}{\underset{︸}{E_{\gamma }\left(=\delta_{0}+\delta_{1}e_{1}+\cdots +\delta_{n}e_{n}\right)}}+\underset{white\;noise}{\underset{︸}{\left(u_{0j}^{\prime }+r_{ij}^{\prime }\right)}}}} \end{array}$$

The spatial filtering component functions as an additional proxy variable accounting for the spatial effect in the data. As a result, the spatial autocorrelation of random effects in the model are accounted for, and corresponding white noise is independent and identically distributed (i.i.d.). It should be noted that eigenvector filtering in this model is applied to level-2 (county level). Therefore, the number of available eigenvectors is *n*, which is the number of areal units in level-2.

All statistical analyses and figures in this research were made using statistical software R 3.4.2 (R Core Team, Vienna, Austria), and the maps were visualized using ESRI ArcGIS 10.4 (ESRI, Redlands, CA, USA). The *glmer* function in the *lme4* package of R was used to estimate the MGLMs [34]. The *ME* function in the *spdep* package in R was used to search the eigenvectors from the spatial lag variant in a GLM framework that provides the spatial lag process [36].

## 3. Results

### 3.1. Spatial Filtering Process

**Figure 4:**
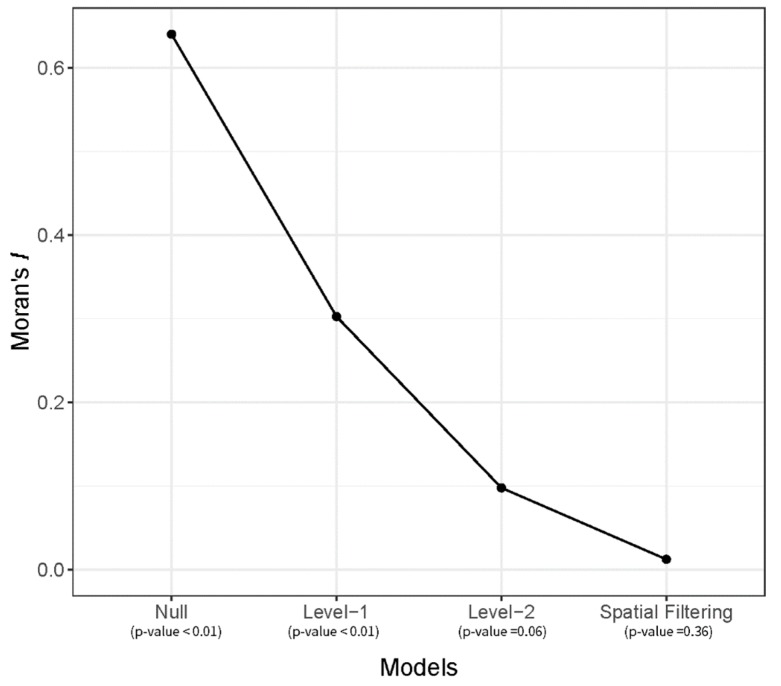
Spatial autocorrelation in random effects of multilevel models.

Figure 4 presents the changes in spatial autocorrelation as different levels of the multilevel model, and eigenvector spatial filtering is applied. The null model shows that Moran’s *I* of random effects is 0.64 (*p*-value ≅ 0.0), which is a strong positive spatial autocorrelation. The random effect of level-1, $e_{ij}$ (in Equation (1)), shows a positive spatial autocorrelation with Moran’s *I* value at 0.30 (*p*-value < 0.001). Furthermore, the random effect of level-2, $u_{0j}+e_{ij}$, shows a positive spatial autocorrelation with Moran’s *I* value at 0.10 (*p*-value = 0.06). This indicates that the multilevel model in this study requires eigenvector spatial filtering.

**Figure 5:**
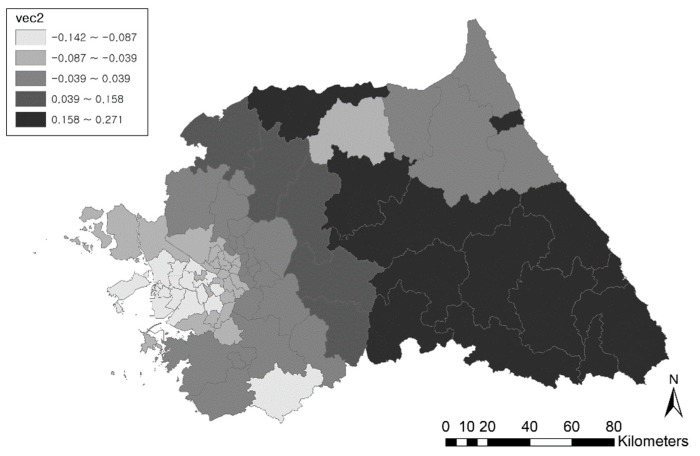
Spatial pattern of eigenvector no. 2.

By applying the spatially filtered multilevel model, eigenvectors were extracted from the spatial lag process, and one eigenvector was selected, eigenvector no. 2. Comparing all generated eigenvectors, eigenvector no. 2 decreased the largest amounts of Moran’s *I* value. If additional eigenvectors are added to the multilevel model, the corresponding Moran’s *I* value becomes negative. The Moran’s *I* value, 0.01 (*p*-value = 0.36), in the random effects of the spatially filtered multilevel model shows that the positive spatial autocorrelation in the model becomes negligible, having no significant effect in the model estimation. The spatial pattern of the selected eigenvector is displayed in Figure 5, and the clustered pattern is noticeable in the map.

### 3.2. Modeling Results

Table 2 presents the analysis results. The null model tests whether multilevel modeling is necessary for the analysis [35]. The intraclass correlation coefficient (ICC) shows the level of variance of the dependent variable explained by between-group variance in a model. Consequently, it shows whether multilevel modeling is required or not. In this malaria occurrence analysis, the ICC of 0.664 implies that between-group variance accounted for 66.4% of malaria occurrences and within-group variance accounted for 33.6%. This result shows that applying multilevel analysis for the malaria occurrence data is required.

The level-1 model applies only to district-level variables, assuming that the county-level effect is negligible in the analysis. Given the AIC (Akaike information criterion) value, the level-1 model result shows a better estimation compared to the null model. Among the level-1 variables in the model, three variables (average age, rice paddy ratio, and distance from the DMZ) are significant.

The level-2 model added the level-2 (county level) variables to the level-1 model. Two level-2 variables (health budget per capita and military base area ratio) and three level-1 variables (average age, rice paddy ratio, and distance from the DMZ) are significant in this model. The variance of random effects in level-2 decreases to 0.25, and the AIC value decreases as well. This indicates that the level-2 model better estimates the malaria occurrences compared to the level-1 model.

In the spatial filtering model, four level-1 variables (housing costs, average age, rice paddy ratio, and distance from the DMZ) and two level-2 variables (health budget per capita and military base area ratio) are significant in the model. The housing cost variable is significant only in the spatial filtering model. The variable coefficient signs remain constant in the level-2 model and the spatial filtering model. The AIC value in the spatial filtering model decreased from 1882.0 to 1879.3, showing improved model estimation. The group-level variance decreased from 0.25 in the level-2 model to 0.20 in the spatially filtered model. This indicates that the spatial filtering component accounts for the group-level variance. These results show that the spatially filtered multilevel model yields the most appropriate estimation in this study.

### 3.3. Results Interpretation—Spatially Filtered Multilevel Model

The results indicate that spatial units with lower housing costs, lower average age, higher rice paddy ratios, less distance from the DMZ, lower health budget per capita, and higher military base area ratios are more susceptible to malaria risks.

Military base areas show the unique characteristics of malaria occurrences in Korea. Because malaria’s re-emergence originated in military servicemen in the DMZ, military patients have played a major role in malaria transmission in Korea [7]. Malaria transmission in Korea is unique for the following reasons. First, because it was initiated from adjoining North Korea, malaria transmission in South Korea was hardly controlled because of the political conflict between North and South Korea. Due to the continued confrontation, most troops stationed in the DMZ are easily exposed to a high risk of malaria [31]. Second, military activities in the DMZ such as overnight patrols leave military servicemen easily exposed to malaria-carrying mosquitoes [29]. Third, limited accessibility to the DMZ makes it difficult for health authorities to find potential patients among the military servicemen and appropriately treat them [30].

Along with the military base area, the distance from the DMZ is a variable that explains the unique characteristics of malaria in Korea. It reflects the general transmission mechanisms of vector-borne diseases whereby people in adjacent areas are more likely to be infected than those in distant areas. The distance from the DMZ can be interpreted as the level of malaria influence from North Korea as well, since malaria’s re-emergence in South Korea originated from and is affected by North Korea [27]. Currently, the DMZ is a barrier preventing South Korea’s disease control efforts [7]. However, the vector mosquitoes from North Korea can freely cross the border and infiltrate.

Housing cost represents the economic status of the region and provides spatial patterns for the disparity in Korea as well [2]. It is highest in Seoul, the capital city, and becomes lower as further away from Seoul. Most malaria occurrences are concentrated near the DMZ, which is distant from Seoul. The spatial distribution of the disease implies that areas with lower housing costs are more susceptible to malaria risks.

Average age is applied to explain the effect of age on malaria risks. Young adults in their 20s were most susceptible to malaria from 2001 to 2011 in Korea [16]. Most military servicemen stationed in the border area are in their early 20s. Furthermore, the residents of high-risk areas, including Incheon, Goyang, and Gimpo, as common urban areas, tend to have a lower average age than other rural areas.

The rice paddy ratio is significant in explaining the relationship between malaria transmission and human activities. Considering the life cycle of the vector mosquitoes that lay their eggs in stagnant water, rice paddies are ideal places for mosquito breeding [37]. In practice, more than 50% of the larvae of *Anopheles sinensis*, which are prime vector species of malaria in Korea, were found in rice paddy areas according to the research conducted in Ganghwa and Paju [25]. The western parts of Seoul’s metropolitan area, including Goyang, Paju, and Gimpo, are mostly plains and rice paddy areas. Such land use may lead to prominent malaria transmission in these areas.

Health budget per capita reflects the service level of medical and health care and its correspondence to malaria occurrences. Generally, a lower health budget per capita implies a higher demand for health care. The results indicate that regions with a low health budget are more susceptible to malaria risks. A good medical care system in South Korea, featuring a public health insurance system and easy access to medical facilities, has prevented malaria from causing any mortality in the country [28]. However, the health budget per capita variable shows that malaria transmission and its influence is significantly affected. Further research using more variables is necessary to determine the effect of the health care system on malaria occurrences in developed countries, which must be different from developing countries with higher malaria risks.

## 4. Conclusions

This study explains the spatial determinants of district- and city-level factors on malaria occurrences by applying a multilevel and spatially filtered multilevel model. The spatially filtered multilevel model improves the ordinary multilevel model by adding spatial filter term accounting for the spatial autocorrelation in the level-2 random effect. The spatially filtered multilevel model appears to be the most appropriate, judging from the AIC and random effect Moran’s *I*. The result presents four level-1 variables (housing costs, average age, rice paddy ratio, and distance from the DMZ) and two level-2 variables (health budget per capita and military base area ratio) that are statistically significant. Malaria in Korea has unique epidemiological characteristics due to political and social circumstances. Imported from North Korea, malaria occurrences in South Korea were initiated and transmitted by military servicemen in the DMZ. Malaria transmission in South Korea has been continuously influenced by adjoining North Korea since then, which can be verified by the distance from DMZ and military base area ratio variables. This study is expected to contribute to further understanding of the mechanisms of malaria transmission and other vector-borne diseases.

This study has limitations as follows: the dependent variable used in this research does not have detailed information about patients such as age, occupation, and sex, which could be significant information for explaining the epidemiological features of malaria. Additionally, the patient address and the location of actual infection are not always identical because of the mobility of the patients and the incubation period of malaria.

## Figures and Tables

**Table 1:** Descriptive statistics and references for the variables in the research. DMZ: demilitarized zone.

Categories		Variables	Unit	Mean	Standard Deviation	References
Dependent variable		Malaria occurrences	Count	0.376	1.415	
Independent variables	District level (Eup, Myeon, and Dong)	Housing cost	1000	1580.0	1360.3	[2,9,10,11,12,13,14,23]
		Sex ratio	-	1.019	0.092	[2,9,12,14,16]
		Average age	-	41.26	4.559	[10,13,14,15,16]
		Rice paddy area ratio	%	4.1	8.756	[15,24,25]
		Distance from DMZ	km	4.482	2.663	[7,24,26,27,28]
	City and county level (Si, Gun, and Gu)	Health budget per capita	\	47,337	29,278	[2,11,13,14]
		Military base area	%	16.78	25.21	[7,24,26,27,29,30]

**Table 2:** Estimation results of the models.

Categories	Multilevel Models			Spatially Filtered Multilevel Model
	Null Model	Level-1 Multilevel	Level-2 Multilevel	
Fixed effects				
**Level-1 Variables**				
Housing costs	-	−0.03	−0.17	−0.24 *
Sex ratio	-	0.06	0.07	0.07
Average age	-	−0.25 **	−0.21 **	−0.20 **
Rice paddy ratio	-	0.15 **	0.15 **	0.15 **
DMZ distance	-	−1.31 **	−1.31 **	−1.20 **
**Level-2 Variables**				
Health budget per capita	-	-	−0.91 **	−0.82 **
Military base area	-	-	0.22 **	0.32 **
Random effects				
Group-level variance	1.74	0.50	0.25	0.20
Constant	−1.94	−2.06	−2.27	−2.31
AIC	1998.8	1901.5	1882.0	1879.3
Log-likelihood	−997.4	−943.8	−932.0	−929.6
Moran’s *I* of random effect	0.64 **	0.30 **	0.10 (*p* = 0.06)	0.01

* *p*-value < 0.0; ** *p*-value < 0.001; AIC: Akaike information criterion.
